# Ultrasonication as a tool to develop starch nanoparticles from macrophytes to tailor starch properties

**DOI:** 10.1016/j.ultsonch.2025.107711

**Published:** 2025-12-06

**Authors:** Romee Jan, Adil Gani, Asima Shah, Irfan Ahmad Raina, Asir Gani

**Affiliations:** a Department of Food Science and Technology, University of Kashmir, Hazratbal, Srinagar, J & K UT, India; b School of Bioengineering and Food Technology, Shoolini University, Solan, H.P, India

**Keywords:** Macrophyte starch, Ultrasound-assisted processing, Starch nanoparticles, Physicochemical analysis, Sustainable biomaterials

## Abstract

Starch is a popular type of biopolymer that has multiple uses in industry and there is an increased interest in searching the possible sustainable sources in underutilized plants. The paper was aimed at extracting and characterizing starch from macrophyte narrowleaf cattail (*Typha angustifolia*) and lotus (*Nelumbo nucifera*) of Dal Lake, Kashmir. The two species possessed high starch content of 67.11 ± 1.03 % and 66.18 ± 1.04 % with moderate amylose content of 13.67 ± 0.70 % and 13.45 ± 0.11 %, respectively, indicating their alternative use in the food industry. Proximate analysis showed that cattail starch contained more protein (6.28 ± 0.55 %) and fat (2.15 ± 0.03 %) than lotus, indicating that the two species are functionally different and may be applied in specific functional applications. Ultrasonic processing, a green efficient method was used where the acoustic cavitation by high frequency sound waves improved mass transfer and destruction of plant cell walls, leading to structural changes in the extracted starches thereby enhancing the functional and physicochemical properties. Ultrasonic nano-reduction minimized particle size and improved the colloidal stability of the particles as shown by the low polydispersity index and high negative zeta potential. Such physicochemical improvements would increase starch functionality in biodegradable packaging, pharmaceutical, and food technology applications. This study identifies the untapped potential of macrophytes from Dal Lake as renewable sources of biodegradable starch, encouraging the sustainable use of local biodiversity and valuable addition products, which boosts the economic growth of the area.

## Highlights

• Starch from *Typha angustifolia* and *Nelumbo nucifera* showed approx. 67% yield.
• Ultrasonication reduced starch size to nanoscale, enhancing uniformity.
• Nano-starch showed lower PDI and higher zeta stability.
• Underutilized Dal Lake macrophytes serve as renewable starch sources.
• The research outcome supports sustainable and eco-friendly material development.

## Graphical abstract

**Figure:**
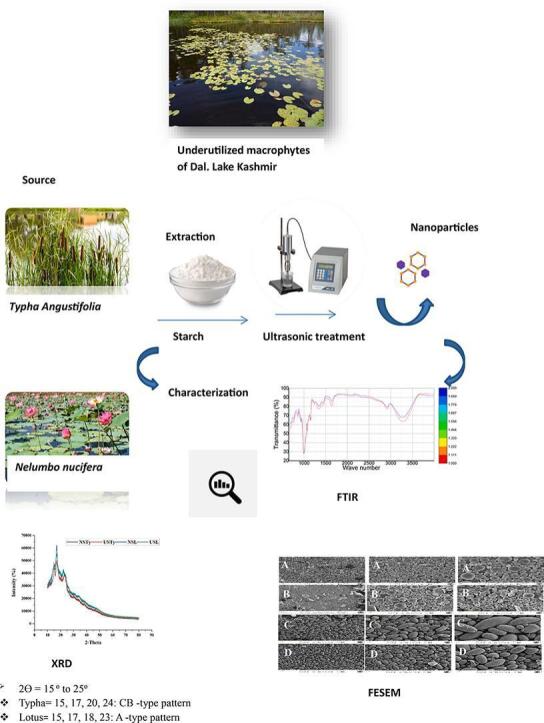

## 1 Introduction

Dal Lake in the Kashmir Valley is not only an important ecological resource but also a storehouse of various aquatic macrophytes. These macrophytes play a crucial role in maintaining the lake’s ecological balance; however, despite their abundance and biochemical richness, they remain largely underutilized from a biotechnological and industrial perspective. In fact, species such as *Typha angustifolia* (*T. angustifolia*) and *Nelumbo nucifera* (*N. nucifera*) proliferate rapidly and are periodically removed as part of lake conservation programs to control eutrophication and maintain water quality [1]. The use of biomass obtained from such routine management practices ensures that the material source is ecologically sustainable, preventing overharvesting and contributing to the ecological restoration of the lake. The biochemical composition of these macrophytes has been recently featured, with a high carbohydrate content being pointed out, which highlights their potential as alternative sources of starch [2]. The use of such underexplored plant sources to extract starch has gained increasing attention in view of the growing demand for sustainable and environmentally friendly raw materials. Starch, a biodegradable polymer has been investigated in several ways such as its nano-reduction to amplify its functional attributes. Isolated starch of freshwater macrophytes shares a lot of similarities with the ones of rice, potato, and maize in terms of their industrial potential [3]. Macrophyte-derived starches, however, often possess relatively large granules, high crystallinity, and lower solubility compared to conventional starches, posing challenges in their direct industrial application. Ultrasound treatment has emerged as a promising, eco-friendly technique for starch modification and nano-reduction, as it induces cavitation-based disruption of granules, enhances solubility and surface reactivity, and improves functional performance [4], [5].

Nano-reduced starch has its peculiarities, including enhanced solubility and enhanced surface area, which makes it suitable in the field of drug delivery systems as well as biodegradable films and as the stabilizer in food products [6]. Kundangar & Abubakar [1] established that the major occupant of the littoral areas along the eastern and southern areas of Dal lake is Cattail. A range of aquatic macrophytes such as *T. angustifolia* (narrowleaf cattail) has been found as a possible source of starch. *N. nucifera*, popularly referred to as the sacred lotus, is a water plant that is widely planted in areas such as India, China and Japan. Although its starch content in seeds and rhizomes has received significant research attention, stems have recently become the focus of research as a source of starch. Lotus has a promising future in the industrial world because the stem starch has special physicochemical properties [7]. The starch obtained from these plants can undergo nano-reduction processes like the mild treatment of alkali hydrolysis and ultrasonic to form starch nanoparticles. Such nanoparticles can be described in terms of thermal, morphological, and functional characteristics, which points to their potential in many industrial applications [8].

This research seeks to discuss the extraction of starch in selected macrophytes of Dal Lake, such as cattail and lotus and their subsequent nano-reduction. The work will compare physicochemical characteristics of the extracted starch and the nanoparticles that were obtained and find their suitability in food technology, bioplastics and other industrial fields. This study does not only add value to the sustainable use of aquatic resources, it also provides information on the creation of value products using underutilized plant materials. The process of extracting starch using unusual materials complies with the increased need in eco-friendly and sustainable materials. A biodegradable polymer such as starch has been studied on its possible uses in different applications, such as reduction of the polymer to nano-scaffolds to improve the functionality of the material. Nano-reduced starch has distinctive properties, i.e. higher solubility and larger surface area, which has a place of implementation in drug delivery systems, biodegradable films, and as stabilizers in food products [9].

Research on starch obtained in freshwater macrophytes such as cattails and common reed have been carried out but are comparatively scarce [10], [3], [11], [12]. Although local populations utilize them, the traditional application of these plants is not well reported in the scientific literature; thus, the aim of the present study is to extract starch of the selected plants of Dal Lake (cattail and lotus) and nano-reduce them. The study aims to compare the physicochemical characteristics of the extracted starch and the resulting nanoparticles to evaluate their functional enhancement, with particular emphasis on applications relevant to food formulation and biodegradable film development. This research work does not only add value to the sustainable use of aquatic resources, it also provides information on the creation of value products using underutilized plant materials.

## 2 Materials and methods

### 2.1 Materials

The samples Narrowleaf cattail (*Typha angustifolia*) were gathered from Dal Lake, Hazratbal, Srinagar and indigenous variety of lotus (*Nelumbo nucifera*) were obtained at the market sites along the banks of Dal Lake, Srinagar, Jammu and Kashmir, India. The stems and rhizomes were scraped to remove the adhering soil, added with cold water and chopped into tiny fragments for the extraction process. The utilized reagents and chemicals were of analytical grade.

### 2.2 Extraction of starch

Starch extraction from rhizomes of narrowleaf cattail was done using alkaline steeping method [13]. Rhizomes were wet ground using double distilled water up to ten times (w/v) to create aqueous slurry; pH was kept at 9.5. Constant mixing of slurry was continued until 1 h and then filtered (sieve) to separate fiber particles (72-m sieve). The filtrate was centrifuged at 3000 g, 15 min, decanted and scraped off the upper layer and acquired the upper layer. Sediment was centrifuged once again in the double distilled water. This washing procedure was repeated thrice; the obtained starch was dried in the hot air oven at 40 °C 12 h, and then stored. The starch from lotus stem was obtained by alkaline wet-milling process using the procedure of Noor et al. [14]. The stems were first chopped and homogenized in a chilled, dilute alkali solution (0.1 % sodium hydroxide) using a blender. The resulting slurry was passed through a 100 µm nylon mesh to separate fibrous residues. To ensure complete release of starch granules from the fibers, the pulp was re-ground twice. The filtrate was thoroughly washed with distilled water, and starch was collected by centrifugation at 3000 × g. The yellowish, gummy layer formed on the surface of the starch sediment was carefully removed and discarded. The purified white starch was then rinsed once with ethanol, filtered through Whatman No. 4 filter paper (Whatman, UK), and dried at 40 °C.

### 2.3 Yield of starch

The yield of starch was estimated by the procedure outlined by Awolu & Olofinlae [15] in which yield is defined as the ratio percentage of the weight of starch extracted to the weight of the sample taken.

(1) $$\mathrm{Starch}\;yield\;\left(\%\right)=weight\;of\;starch\;(g)/weight\;of\;sample\;\left(g\right)\times 100$$

### 2.4 Ultrasonication of extracted starch

Native starch (10 g, db) of cattail and lotus was weighed and nano-reduced by ultrasonication method. Starch suspension in water (2 %) was sonicated using the ultrasonic probe (Cole-Parmer, 0471135) with 60 % amplitude, 20 kHz frequency and 150 W power within 10 min. The sonicated suspension was freeze dried (Buchi-lyovapor L- 200) over 12 h and the formed nanoparticles then stored in air tight containers for further use. The ultrasound conditions used for starch size reduction, including power, frequency, and treatment time, were determined based on preliminary trials and insights from recent studies on comparable starch systems. These selected parameters were found to achieve effective particle size reduction and enhanced functional properties while minimizing the risk of significant starch granule degradation [16], [17].

### 2.5 Particle size and zeta potential measurement

A particle size analyser (Litesizer, 500, Anton Paar, Austria) was used to measure the average particle size and zeta potential of the native and nano reduced starch particles. 1/100 of the sample was dispersed in deionized water (Elix-10, Millipore, Molsheim, France) and sonicated in bath sonicator during 20 min at a frequency of 40 kHz, temp. of 25 °C and at neutral PH for proper dispersion of the starch particles.

### 2.6 Starch physicochemical properties

#### 2.6.1 Proximate composition and apparent amylose content

The parameters like moisture, protein, fat, fiber, ash and total starch of the samples were examined as per AOAC [18] as per the protocol followed by Jan et al. [19]. Amylose content of the samples was examined by the method of Morrison and Laignelet [20] and following formula was used for the calculation of amylose content.

(2) $$\mathrm{Amylose}\;Content\;\left(\%\right)=\;28.414\times \;Blue\;value-6.218$$

where blue value (BV) is the absorbance at 635 nm of starch and I_2_/KI solution

#### 2.6.2 Color determination

Lightness (L) was measured as a color value of the starches with the help of Hunter colorimeter (Model I5 Green Macbeth, USA). Whiteness index (WI) was computed as per the equation given by Hsu et al. [21] as follows:

(3) $$\mathrm{WI}=100-\surd \;\left(100-L^{2}\right)+a^{2}+b^{2}$$

where, a, b, and L were Hunter L, a, and b values.

#### 2.6.3 Oil absorption capacity (OAC) and water absorption capacity (WAC)

Water absorption capacity (WAC) and oil absorption capacity (OAC) of the samples was measured in a dry state by pouring 2 g of samples in the centrifuge tube with pre-weight and mixing the mass in the centrifuge tube with 25 mL of distilled water/ mustard oil. The tubes were roused 30 min at 25° C. They were then centrifuged at 5000 rpm with a 15-minute centrifugation (5810R, Eppendorf, Hamburg, Germany). The supernatant was discarded and tubes weighing was done again. Gain in weight was presented as percentage of water/oil absorption.

#### 2.6.4 Swelling power and water solubility

The swelling power and solubility of starches was done according to procedure of Jan et al. [19] (2016) with slight modifications. They were established within 55–95 °C temperature. Centrifuge tubes with starch slurry (2 g/100 mL starch, dry basis) were heated at 55, 65, 75, 85, and 95 °C over 30 min. Cooled tubes were centrifuged at 112 g, 20 min (C24, BL/s. M/s. Remi Laboratory Industries, Mumbai, India). The supernatant was decanted very carefully in petriplates and evaporated and dried at 105 °C over 5 h till it reaches constant weight and weighed to determine the solubility (g/100 g). Swelling power was estimated by weighing the residue. Swelling power and solubility was determined as:

(4) $$\mathrm{Swelling}\;\mathrm{Power}\;\left(g/g\right)=\frac{Weight\;of\;sediment\;paste\;\left(g\right)}{Weight\;of\;sample\;\left(dry\;basis,\;g\right)}$$

(5) $$Solubility\;\left(\frac{g}{100\;g}\right)=\frac{Weight\;of\;soluble\;\left(g\right)}{Weight\;of\;sample\;\left(dry\;basis\right)}\times 100$$

### 2.7 Infrared ATR-Fourier transform spectroscopy

The structural properties of the native and sonicated starch samples were determined using FTIR spectrometer system (Cary 630 FTIR, Agilent Technologies, USA) fitted with an attenuated total reflectance (ATR) accessory. About 2 mg of each dried starch sample was carefully positioned on the diamond ATR crystal, and spectra were recorded within the wavenumber range of 4000 to 400 cm^−1^ at a resolution of 4 cm^−1^. Each spectrum represented the average of 32 scans to enhance signal clarity. A background scan was acquired prior to each sample measurement to minimize atmospheric noise. The spectra were subjected to baseline correction and normalization for comparative evaluation. The major absorption peaks were analyzed to determine the functional groups and to elucidate structural changes induced by the ultrasonication process.

### 2.8 Thermal characteristics

Thermal properties of nano-starch samples were assessed using the differential scanning calorimeter (DSC-1 STARe System, MettlerToledo). 3.5 mg of starch samples were weighed in platinum pans after which 8 μL of deionized water was added to it and placed in the instrument chamber at room temperature. In order to manage the surrounding local environment the N_2_ gas was used in the purge line. The temperature was raised up to 240 °C at a steady temperature of 10 °C/min. An empty platinum pan was taken as a reference.

### 2.9 Scanning electron microscopy (SEM)

Morphological study of the starch samples was performed according to Jhan et al. [13] (2020) under the vacuum using scanning electron microscope (Zeiss EVO 50). Samples were wrapped with adhesive tape attached to a circular stub composed of aluminium and covered with gold vertically. The samples were photographed then with a digital image analyser at an accelerator potential of 20 kV.

### 2.10 X-ray diffraction (XRD)

XRD patterns of native and nano-reduced starch samples were analysed by X-ray diffractometer (X'Pert PRO PANalytical, Netherlands) as per the procedure of Jhan et al. [13]. The diffraction operation was performed at voltage 40 kV, x-ray line 1.5418 A and current 35 mA using the powdered samples on aluminium plate. The diffractograms were obtained at 25 °C, 20 °C, and 15 °C with a step of 0.02/min at 25 °C.

### 2.11 Statistical analysis

Statistical analysis of results (mean values, standard deviation, ANOVA) was determined using commercial statistical package, SPSS (IBM statistics 22). The experiments were conducted in triplicates on dry weight basis and data were then evaluated with the Duncan tests at 5 % level of significance Jhan et al. [13]*.*

## 3 Results and discussion

Understanding the compositional, structural, and functional characteristics of starch is vital for evaluating its suitability in food and industrial applications. These properties influence its behavior during processing and determine its efficiency as a thickening, stabilizing, or biodegradable agent. Ultrasonication, an eco-friendly and non-thermal modification method, can significantly alter starch functionality through cavitation and shear forces, leading to granule disruption, particle size reduction, and molecular rearrangements. Such modifications affect solubility, swelling, and thermal properties. Therefore, investigating the effects of ultrasonication on starches from cattail and lotus provides insight into structure–function relationships and their potential for improved industrial applications. The results have been presented and discussed in the following sub-sections.

### 3.1 Composition, total starch and apparent amylose content (AAC)

Narrowleaf cattail showed a starch content of 11.78 ± 0.11 % that was lower than lotus stem yielding 18.45 ± 0.50 %, respectively. The seed and rhizome of lotus produced 16.57 % and 14.75 % of starches, respectively [22]. According to the previous findings, *Typha* sp. was typically characterized by low starch content (13.01 to 14.5 %) in comparison with other commercial starches like Zea mays (22.40 %) [3]. The purity of starch samples was determined as 71.81 ± 0.50 % for cattail rhizome starch and 80.58 ± 1.03 % for lotus stem starch. Table 1 summarizes the proximate content of starch harvested in cattail and lotus, total starch, amylose content, moisture, protein, fat, fiber and ash. The total starch content of both cattail and lotus starch samples were insignificantly (*p ≥ 0.05*) similar 67.11 ± 1.03 and 66.18 ± 1.04, respectively. These values indicate that the two sources have significant starch yield as earlier values were reported on aquatic plant-based starches [23]. There was also similarity in apparent amylose content between the two species with cattail having a value of 13.67 ± 0.70 and lotus recording 13.45 ± 0.11 (*p ≥ 0.05*). This medium level of amylose is beneficial in the applications that may need a balance in the functionality like those in the development of biodegradable films, thickeners, and controlled-release systems [24]. The moisture content of the starch samples was determined as 10.85 ± 0.08 and 10.32 ± 0.82 %, respectively, with no significant difference being found between the two samples (*p ≥ 0.05*). These values of moisture are within the acceptable limits of starch powders, which are stable in nature and hence the drying and purification processes used in the extraction process were effective. Reduction of moisture is of paramount importance to reduce the growth of microbes and enzyme degradation in storage to maintain the functional properties of the starch [25]. Protein content in the two starches was found to be very different. Cattail starch had 6.28 ± 0.55 % protein, which was much greater than lotus starch at 4.52 ± 0.05 (*p ≤ 0.05*). Such disparity may be due to an incomplete removal of proteins during starch extraction or to species-specific elevated intrinsic protein levels. Protein presence has the potential to affect starch functionality (gelatinization, emulsification properties, and digestibility) and hence widen application range of cattail starch [26]. The amount of fat content was also found to be quite different in that cattail starch had 2.15 ± 0.03 % fat content, whereas lotus had 1.28 ± 0.32 % fat content (*p ≤ 0.05*). Pasting behavior and thermal properties of the starch could be affected by the presence of lipids, which could occur by the formation of amylose–lipid complexes. These complexes are reported to alter gelatinization as well as retrogradation properties, which can be either beneficial or restrictive in accordance with the targeted industrial application [23]. No fiber was detected in either sample of the starch, which indicates that the purification and elimination of non-starch polysaccharides were performed effectively. The low ash content and the same of both samples indicated low mineral contamination (0.03 ± 0.0 %) and this is beneficial in food grade starch processing where high purity is demanded [25]. These results concur with earlier report that indicates non-starch constituents differ with plant species and extraction process [23], [26].

**Table 1:** Composition, total starch and apparent amylose content (AAC) of starch samples.

Samples	Total starch %	AAC %	Moisture %	Protein %	Fat %	Fibre %	Ash %
Cattail	67.11^a^ ± 1.03	13.67^a^ ± 0.70	10.85^a^ ± 0.08	6.28^a^ ± 0.55	2.15^a^ ± 0.03	0.00 ± 0.0	0.03^a^ ± 0.0
Lotus	66.18^b^ ± 1.04	13.45^a^ ± 0.11	11.32^a^ ± 0.82	4.52^b^ ± 0.05	1.28^b^ ± 0.32	0.00 ± 0.0	0.03^a^ ± 0.0

Results are expressed as mean values ± standard deviation of three observations. Means in rank with different superscripts differ significantly (*p ≤ 0.05*).

### 3.2 Colour and whiteness indices of native and sonicated starch

Table 2 displays the colour properties (L*, a*, b*) and whiteness Index (WI) of native and ultrasonated cattail starch (NSC, USC) and lotus starch (NSL, USL). L* value and WI of the starch in lotus stem (86 ± 1.50 and 87.13 ± 1.08) were greater than that of cattail starch (84 ± 1.50 and 83.05 ± 1.08), respectively. These values were a bit smaller than those of lotus stem starch (87.58 %) identified by Naseem et al. [27]. L* value decreased significantly (*p ≤ 0.05*) for starch from 84 ± 1.50 to 81.6 ± 1.10 Cattail and 86 ± 1.50 to 79.5 ± 1.05 lotus after ultrasonication. This reduction in lightness is possibly related to structural changes of starch granules in response to ultrasonic waves, which may lead to aggregation or surface morphology alteration that favour lower reflectance of light [28]. Concerning a* value, the axis of red-green colour, a slight but statistically significant decline was found following sonication in both types of starch. In cattail, a* value changed from 0.4 to 0.3 ± 0.003 and in lotus from 0.6 to 0.3 ± 0.004. Even though the difference was minor, it shows a tendency to switch to less colorful tones in sonicated starch samples, which could be due to the breaking down of pigmenting compounds or some insidious processes of the Maillard process during the ultrasonic procedure [29]. The b* value of the yellow-blue axis was significantly (*p ≤ 0.05*) higher for cattail (5.6 ± 0.21) and lotus starch (7.6 ± 0.21) after ultrasonication. This is an indication that sonication increases yellowish coloration; probably due to the exposure of the chromogenic groups, or the creation of degradation products, which have yellow coloration as the starch structure becomes perturbed [30]. Whiteness Index (WI) which is one of the major variables that reflect visual purity also demonstrated a considerable decrease following the ultrasonic treatment. The WI of NSC was 83.05 ± 1.08 that decreased to 78.4 ± 1.30 in USC and NSL decreased to 81.7 ± 1.30 in USL. These results confirm the observed changes in colour values and indicate that ultrasonication affects the visual quality of starch that could affect its suitability in food formulations where it should be highly whitened [24]. Such findings are consistent with past studies that showed that ultrasound causes granule fragmentation, molecular restructuring, and exposure of internal chromogenic compounds [28], [24]. Sonication may cause the exercise of enzymes such as polyphenol oxidase and, therefore, browning and a decrease in L* values [31]. Sonication may also cause the crystalline structure of starch grains to be disrupted, which results in light scattering and absorption.

**Table 2:** Colour (L, a, b) values and whiteness index (WI) of starch samples.

Samples	NSC	USC	NSL	USL
L*	84^b^ ± 1.50	81.6^c^ ± 1.10	86^a^ ± 1.50	79.5^d^ ± 1.05
a*	0.4^b^ ± 0.003	0.3^c^ ± 0.003	0.6^a^ ± 0.004	0.3^c^ ± 0.004
b*	3.8^d^ ± 0.20	5.6^b^ ± 0.21	4.5^c^ ± 0.10	7.6^a^ ± 0.21
WI	83.05^b^ ± 1.08	78.4^d^ ± 1.30	87.13^a^ ± 1.08	81.7^c^ ± 1.30

Results are expressed as mean values ± standard deviation of three observations. Means in rank with different superscripts differ significantly (*p ≤ 0.05*). NSC: native starch cattail; USC: ultrasonicated starch cattail; NSL: native starch lotus; USL: ultrasonicated starch lotus.

### 3.3 Particle size, polydispersivity index (PDI) and zeta potential

Table 3 presents the size of the particles, polydispersity index (PDI) and the zeta potential of the native and ultrasonated cattail starch (NSC, USC) and lotus starch (NSL, USL). Another significant effect of using ultrasonic processing for extracted starches was a significant decrease in the average hydrodynamic diameter. The native cattail starch (NSC) had a particle size of 88.048 ± 1.10 µm and it was reduced to 756.66 ± 1.50 nm after sonication (USC). Similarly, the particle size of lotus starch (NSL) decreased from 70.674 ± 2.10 to 815.16 ± 1.20 nm (USL) after ultrasonication. Such findings reveal the effectiveness of ultrasound treatment in degrading starch particles into smaller particles, which is probably due to the cavitation effects that produce high shear forces and localized elevated temperatures [28]. The obtained results indicate that ultrasonication not only decreased the average particle size but also the increase the homogeneous distribution of the particle size profile, which is useful to enhance the dispersion stability and performance of food, pharmaceutical, and material applications [29]. The values of PDI indicate the amount of size variation in the sample. A wide distribution of sizes of particles was observed with native cattail starch (NSC) with a high PDI of 3.85 ± 0.03. The PDI decreased significantly after ultrasonication (USC) to 0.49 ± 0.05 which indicates a more homogenized mixture of particles. Likewise, lotus starch (NSL) had PDI equal to 1.230 ± 0.11 and in the case of USL the same was found to be 0.98 ± 0.20 via sonication. A decreased PDI signifies an evenly distributed particle size which is essential for reproducibility, and stability in emulsions or nano-delivery systems [24]. Zeta potential measurements give an insight into the stability of dispersions of starch. The native Cattail starch (NSC) exhibited zeta potential values of −12.78 ± 0.03 mV and, upon ultrasonic treatment, the values changed to −29.3 ± 0.02 mV (USC). Likewise, the zeta potential of NSL was −12.36 ± 0.50 mV which changed to –23.2 ± 0.50 mV in USL. This rise in negative surface charge is presumably caused by the exposure of ionizable groups that is caused by the disruption of the granule structure during the ultrasonication. This leads to the increase of the electrostatic repulsion between the particles, which boosts dispersion stability and decreases the aggregative propensity [28], [30]. Ultrasonication evidently had an effect on the physicochemical characteristics of cattail and lotus starches. The large decrease in particle size, PDI and more negative zeta potential is indicative of enhanced stability and uniformity of the starch dispersions. These changes are useful in increasing the variety of industrial uses of starches, particularly in food processing, medicines and biodegradable plastics. These results are in line with earlier work that has shown ultrasonic treatment is an efficient approach towards minimizing the size of particles and improving colloidal stability through changing the surface properties of starch granules [28], [24].

**Table 3:** Particle size and zeta potential of starch samples.

Samples	NSC	USC	NSL	USL
Average hydrodynamic diameter	88.048^a^ ± 1.10 µm	756.66^c^ ± 1.50 nm	70.674^d^ ± 2.10 µm	815.16^b^ ± 1.20 nm
Particle size distribution (nm)	73.277^a^ ± 0.50 µm	751.70^c^ ± 0.11 nm	70.674^d^ ± 1.35 µm	777^b^ ± 0.40 nm

PDI (%)	3.85^a^ ± 0.03	0.49^d^ ± 0.05	1.230^b^ ± 0.11	0.98^c^ ± 0.2 nm
Zeta potential (mV)	−12.78^c^ ± 0.03	−29.3^a^ ± 0.02	−12.36^d^ ± 0.05	–23.2^b^ ± 0.50

Results are expressed as mean values ± standard deviation of three observations. Means in rank with different superscripts differ significantly (*p ≤ 0.05*). NSC: native starch cattail; USC: ultrasonicated starch cattail; NSL: native starch lotus; USL: ultrasonicated starch lotus.

### 3.4 Functional characteristics

#### 3.4.1 Water absorption capacity

Water absorption capacity (WAC) is the capacity of starch samples to absorb water, which is a key functional characteristic that determines viscosity in processing. Increase in WAC is usually related to an increased surface area of starch nanoparticles that increases their water-retention capacity [8]. Table 4 shows the WAC and OAC of native and sonicated of cattail (NSC, USC) and lotus (NSL, USL) starch samples. The findings reveal that the starch nanoparticles (USC and USL) always had a greater WAC in comparison to their native starches (NSC and NSL). Ultra-sonicated starch of cattail (USC) showed a significant (*p ≤ 0.05*) increase in both WAC and OAC (478 ± 1.10 g/100 g and 475.1 ± 1.30 g/100 g) relative to sonicated lotus starch (295 ± 1.45 g/100 g and 283 ± 1.20 g/100 g), showing a better hydrophilic and lipophilic behavior after the sonication treatment. It is worth noting that USC exhibited the best WAC (478 ± 1.10 g/100 g) and OAC (475.1 ± 1.30 g/100 g) values followed by the USL (WAC: 295 ± 1.45 g/100 g and OAC: 283 ± 1.20 g/100 g). This is due to the fact that ultrasonication led to structural disruption of starch granules exposing more hydroxyl groups and surface area thus increasing the interaction of starch with both water and oil [32]. In line with these observations, Vela et al*.* [33] indicated that ultrasonic treatment results in cavitation that caused fragmentation and structural damages on starch granules. The net effect of these structural changes is that they enhance the amount of surface available to interaction with water and oil which in turn leads to improved physicochemical properties like swelling power and solubility. Moreover, Prakruthi et al. [34] showed that cavitation and shear forces that are produced during ultrasonication increase the area of starch granules leading to a higher rate of chemical reaction and enhanced functionality.

**Table 4:** Water absorption capacity (WAC), oil absorption capacity (OAC), Swelling power and solubility of starch samples.

Samples	NSC	USC	NSL	USL
WAC (g/100 g)	347.2^b^ ± 2.50	478^a^ ± 1.10	168^d^ ± 1.50	295^c^ ± 1.45
OAC (g/100 g)	337^b^ ± 3.10	475.1^a^ ± 1.30	176^d^ ± 1.11	283^c^ ± 1.20
Swelling power (g/g)				
55 °C	3.6^e^ ± 0.12	7.2^e^ ± 0.25	12.41^e^ ± 0.11b	14.57^e^ ± 1.01
65 °C	3.8^d^ ± 0.5	7.3^d^ ± 0.30	12.63^d^ ± 0.08	14.66^d^ ± 1.20
75 °C	4.1^c^ ± 0.18	7.4^c^ ± 0.09	13.80^c^ ± 0.06	14.88^c^ ± 0.96
85 °C	4.5^b^ ± 0.20	7.5^b^ ± 0.10	13.95^b^ ± 0.08	15.10^b^ ± 0.50
95 °C	4.9^a^ ± 0.11	7.6^a^ ± 0.15	14.09^a^ ± 0.02	15.23^a^ ± 0.11
Solubility (g/100 g)				
55 °C	78.4^e^ ± 1.30	83.05^e^ ± 1.08	81.7^e^ ± 1.30	87.13^e^ ± 1.08
65 °C	80.34^d^ ± 1.30	85.41^d^ ± 1.20	83.29^d^ ± 0.03	89.10^d^ ± 0.11
75 °C	83.57^c^ ± 0.55	88.92^c^ ± 1.10	86.51^c^ ± 0.87	92.66^c^ ± 0.05
85 °C	86.29^b^ ± 0.11	93.68^b^ ± 0.92	89.47^b^ ± 1.10	94.82^b^ ± 0.18
95 °C	88.97^a^ ± 1.45	96.57^a^ ± 0.04	92.08^a^ ± 0.55	97.15^a^ ± 0.50

Results are expressed as mean values ± standard deviation of three observations. Means in rank with different superscripts differ significantly (*p ≤ 0.05*). NSC: native starch cattail; USC: ultrasonicated starch cattail; NSL: native starch lotus; USL: ultrasonicated starch lotus.

#### 3.4.2 Swelling power

Swelling power (SP) of starch samples was measured at a temperature of between 55 °C to 95 °C. Increase in SP was also noted gradually with rise in temperature in all the samples which is in line with the increase in the molecular mobility and rupture of intermolecular hydrogen bonds at high temperatures. Notably, the swelling power of sonicated starch samples (USC and USL) was found much higher than that of native starch samples (NSC and NSL) at any given temperature (*p ≤ 0.05*). The USL sample was found to have the highest swelling power (15.23 ± 0.11 g/g) followed by NSL (14.09 ± 0.02 g/g), USC (7.6 ± 0.15 g/g) and NSC (4.9 ± 0.11 g/g) at 95 ^°^C. (Table 4). This considerable SP for the sonicated sample can be ascribed to the structural changes that are caused by ultrasonication. Among them are the loss of granular integrity and partial fragmentation of starch polymers, which facilitates the increase in penetration of water into the granule matrix and facilitates gelatinization [33]. This augmented swelling behavior was probably due to the greater exposure of hydrophilic sites and a decline in crystalline areas.

#### 3.4.3 Solubility

Solubility of starch samples was evaluated as a parameter of temperature, where there was evident growth of solubility with corresponding rise in temperature. This tendency is also in correspondence with the interruption of hydrogen bonds, existing between molecules at high temperatures, which allows dissolving starch chains in the solution. Sonicated starch samples of cattail and lotus showed a much higher solubility (97.15 ± 0.50 g/100 g and 96.57 ± 0.04 g/100 g), at all temperatures (*p ≤ 0.05*) than their native starch samples (92.08 ± 0.55 g/100 g and 88.97 ± 1.45 g/100 g) (Table 4). The increased solubility of sonicated starches can be attributed mainly to the mechanical influence of ultrasonic energy on crystalline sites of the starch granules that facilitates the release of amylose and amylopectin chains into the solution [33]. This organization of strand is conducive to better contact with water which leads to the high level of solubility and proves the effectiveness of sonication to alter the functionality of starch in the fields of industrial and food applications.

### 3.5 Morphological analysis

**Fig. 1:**
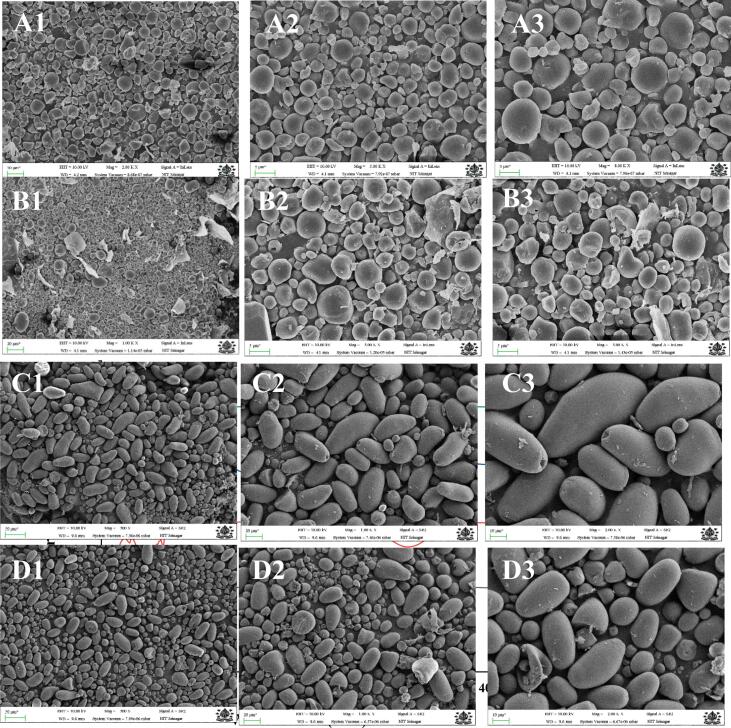
Field emission scanning electron micrographs of liquorice: A1, A2, A3 represent the native starch cattail; B1, B2, B3 represent the ultrasonicated starch cattail; C1, C2, C3 represent the native starch lotus; D1, D2, D3 represent the ultrasonicated starch lotus, respectively.

Surface morphology of native and sonicated starch samples of cattail and lotus samples were observed by Field Emission Scanning Electron Microscopy (FESEM) at different magnifications (Fig. 1). The images are identified as A1-A3, B1-B3, C1-C3, and D1-D3, the samples and magnification levels of the samples. The starch grains of the native cattail species had a morphology mostly spherical with a rather smooth surface and even size distribution. The granules seemed distinct and closely stacked at higher magnification (A3) to reflect a dense crystalline structure of the native starches. The cattail starch samples ultrasonicated presented significant differences in morphology with the native sample. The granules surface was rougher and irregular fragmentation was noticed, mostly at high magnifications (B3). The occurrence of the surface pores and broken edges of the granules indicates that the ultrasonic process caused mechanical destabilization of the starch granules causing the breakdown of the ordered structure. Syed et al. [3] reported that cattail contains small starch granules, mainly polygonal and irregular in shape with few oval ones. Whereas, bigger granules as longitudinal and rod-shaped were found in lotus rhizome. The native lotus starch granules were predominantly oval smooth and with regular shapes and compact appearance at the lower magnification (C1). At magnification (C3), the granules had been shown to retain a uniform surface morphology with no visible damage, which indicated a well-organized and intact crystalline structure. Granules of lotus starch changed their morphological characteristics after ultrasonication. There was erosion, size reduction and fragmentation of the granules, particularly apparent in D3. Many granules looked irregular and collapsed, which suggested that ultrasonication was effective to transform the surface structure and partially destroy the integrity of the granules. The morphological differences noticed between the native and ultrasonicated starch samples validate the fact that ultrasonication has a great impact in disturbing the granular structure of starch. The sonicated material of roughness, fractured edges and pores are in agreement with the cavitation effects and shear forces produced through ultrasonication that promote the breakage of the granules and surface area increase. Such morphological alterations are quite consistent with the functional property improvements that have been measured in this study, including elevated water absorption and swelling power, and solubility. The interrupted framework allows more easy solubility of water and solvents, lowers gelatinization temperatures and enhances reactivity. The results obtained are consistent with earlier studies in which ultrasonication was proven to be an effective method in size reduction of grains and in conferring surface modifications in starch that enhances better functionality in food and industry [33].

### 3.6 FTIR-structural properties

**Fig. 2:**
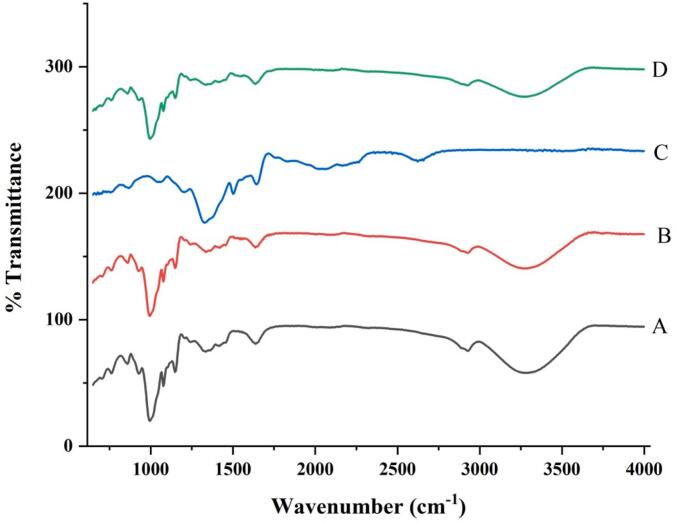
FTIR Spectroscopy characteristics of starches. A: native starch cattail; B: ultrasonicated starch cattail; C: native starch lotus; D: ultrasonicated starch lotus.

The FTIR spectra of starch samples from cattail and lotus both in native and ultrasonicated forms, are shown in Fig. 2. The spectra displayed characteristic absorption bands typical of polysaccharides, confirming the starch identity. For the native cattail starch (A) and lotus starch (C), broad absorption bands were observed around 3200–3400 cm^−1^, corresponding to O–H stretching vibrations, which indicate the presence of hydroxyl groups inherent in starch structure. Peaks around 2922.233 cm^−1^ were attributed to C–H stretching vibrations from –CH_2_ groups. The bands at 1640.029 cm^−1^ correspond to the bending vibration of absorbed water molecules, a common feature in native starches. Upon ultrasonication treatment (B and D), noticeable changes were observed in the FTIR spectra. The intensity of the O–H stretching band slightly decreased, indicating possible disruption of hydrogen bonding and partial depolymerization of starch molecules due to ultrasonic cavitation [35]. Additionally, there was a small shift in peak positions and a slight broadening of the bands in ultrasonicated samples, suggesting structural modifications at the molecular level [36]. The fingerprint region (1200–800 cm^−1^), associated with C–O and C–C stretching vibrations, showed minor but significant differences in peak intensities and shapes after ultrasonication, further supporting the alteration in starch structure such as partial depolymerization and disruption of molecular interactions within the starch granules [8]. These modifications are consistent with the reduction in particle size and enhanced surface area observed in other characterization techniques, such as dynamic light scattering. The above results of FTIR analysis demonstrated that ultrasonication induces subtle yet important physicochemical changes in starch structure, which can enhance its functional properties for industrial applications such as biodegradable films, drug delivery systems, and food additives.

### 3.7 Thermal properties

Differential Scanning Calorimetry (DSC) was used to analyse the thermal behaviour of native and ultrasonated starch samples of cattail (NSC, USC) and lotus (NSL, USL) summarized in Table 5 with the following important parameters being onset temperature (T_O_), peak temperature (T_P_), conclusion temperature (T_C_), and enthalpy change (ΔH). NSC had the higher thermal transition temperatures with T_O_ 68.52 ± 0.30 °C, T_P_ 96.13 ± 1.10 °C and T_C_ 129.45 ± 1.04 °C, and ΔH 3.85 ± 0.03 J/g. Upon ultrasonication (USC), all the thermal parameters were reduced significantly 54.62 ± 0.50 °C (T_O_), 92.59 ± 0.11 °C (T_P)_, 112.31 ± 0.31 °C (T_C)_, and 2.79 ± 0.20 J/g (ΔH). Similarly, the lotus starch samples showed lower thermal transition values following sonication. For lotus starch it deceased from 53.24 ± 0.08 °C (T_O_), 86.15 ± 1.04 °C (T_p_), 125.15 ± 1.10 °C (T_c_), and 13.04 ± 0.06 J/g (ΔH) to 51.43 ± 0.11 °C (T_O_), 83.27 ± 1.05 °C (T_p_), 118.26 ± 1.08 °C (T_c_), and 12.87 ± 0.05 J/g (ΔH), respectively, after sonication. The increased thermal transition temperatures that were found with native samples may be attributed to the higher concentration of intact and bigger starch granules, which necessitate higher thermal energy to break and gelatinize [37]. The large change in thermal parameters following ultrasonication reflects a structural change, such as the disruption of crystalline regions and amorphization of the starch granules partially. The major forces behind these changes are the mechanical impacts of ultrasonication in which cavitation produces shear forces that destroy molecular chains and undermine inter-chain hydrogen bonding [33]. The decreased enthalpy change (ΔH) of sonicated samples indicates that fewer energies are needed for gelatinization and proves the assumption of the weaker crystalline structure and higher levels of amorphous material. These kinds of modifications make starch better in applications requiring lower processing temperatures and paste forming capacity, beneficial in food formulations and in industry where a desired gelatinization is required.

**Table 5:** Thermal characteristics of starch samples.

Samples	T_O (°C)_	T_P (°C)_	T_C (°C)_	ΔH (J g^−1^)
NSC	68.52^a^ ± 0.30	96.13^a^ ± 1.10	129.45^a^ ± 1.04	3.85^c^ ± 0.03
USC	54.62^b^ ± 0.50	92.59^b^ ± 0.11	112.31^d^ ± 0.31	2.79^d^ ± 0.20
NSL	53.24^c^ ± 0.08	86.15^c^ ± 1.04	125.15^b^ ± 1.10	13.04^a^ ± 0.06
USL	51.43^d^ ± 0.11	83.27^d^ ± 1.05	118.26^c^ ± 1.08	12.87^b^ ± 0.05

Results are expressed as mean values ± standard deviation of three observations. Means in rank with different superscripts differ significantly (*p ≤ 0.05*). NSC: native starch cattail; USC: ultrasonicated starch cattail; NSL: native starch lotus; USL: ultrasonicated starch lotus.

### 3.8 X-ray diffraction analysis

**Fig. 3:**
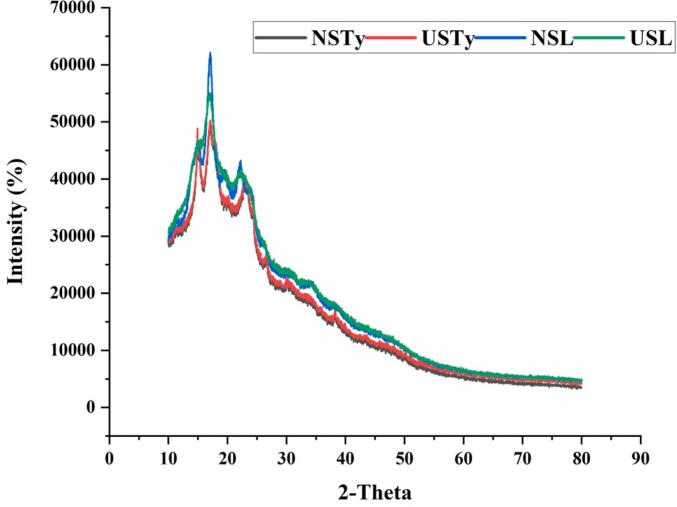
XRD of starch samples. NSC: native starch cattail; USC: ultrasonicated starch cattail; NSL: native starch lotus; USL: ultrasonicated starch lotus.

X-ray diffraction (XRD) was used to examine the crystalline structure of native and ultrasonicated starch samples of cattail (NSC, USC) and lotus (NSL, USL) and the results are depicted in the given diffraction patterns (Fig. 3). In the XRD spectra, there are typical broad diffraction peaks centered at 2θ = 15° to 25° that are characteristic of semi-crystalline starch materials. The cattail starch exhibited diffraction peaks at 15, 17, 20, 24 and displaying CB −type pattern whereas lotus rhizome starch had its peaks at 15, 17, 18 and 23 exhibiting A −type pattern. It is reported that cattail rhizome and pollen starch had CB-type crystallinity with 5, 20 and 24° of crystal peaks (5.96) [3]. The B-type allomorphs were reported to be more than the A-type in the XRD pattern of cattail rhizome [12]. The native samples (NSC and NSL) have sharper and more distinct peaks especially at 17° and 23° which is ascribed to greater crystallinity than the sonicated samples (USC and USL). Conversely, the ultrasonicated samples revealed lower peak intensity and higher diffusion peaks which is indicative of a reduced crystalline order following ultrasonic processing. Such decrease in peak intensity and expansion of the diffraction peaks of the ultrasonicated starch samples suggests the disturbed order of crystalline starch regions, presumably due to the cavitation force and shear force of ultrasonication process. These effects cause fragmentation of starch granules, loss of order, and transformation of the crystalline regions into amorphous structures. The observed reduction of crystallinity in USC and USL samples is associated with better functionalities like enhanced water uptake, solubility and swelling power discussed above in previous sections. These results are consistent with prior research in which ultrasonication has been demonstrated to decrease the starch crystallinity, enhancing the functional properties of starch in food and pharmaceutical purposes [33].

## 4 Conclusions

This research studied the starch extracted from underutilized macrophytes: cattail and lotus, obtained from Dal Lake, Kashmir and their comparison for physicochemical characteristics before and after nano-reduction. The total starch has been found to be very high in both starch sources with moderate amylose content, which implies their alternative use as economically viable substitute to the conventional starch sources in industrial and food application. Ultrasonication-based nano-reduction was a viable method of reducing particle size and enhancing the dispersion stability through the minimization of the polydispersity index and maximization of the negative zeta potential. These modifications will likely improve the functional properties of the starches in the targeted applications, such as in foods, pharmaceuticals, and biodegradable packaging materials. Future studies could focus on systematically optimizing ultrasonication conditions, including power, frequency, and treatment duration, to further improve the functional properties of these starches. Moreover, evaluating their performance in targeted industrial applications, along with assessing their sustainability and economic viability, would help fully establish their potential as alternative starch sources.

## CRediT authorship contribution statement

**Romee Jan:** Writing – review & editing, Writing – original draft, Visualization, Validation, Resources, Methodology, Investigation, Formal analysis. **Adil Gani:** Visualization, Validation, Supervision, Resources, Project administration, Funding acquisition, Data curation, Conceptualization. **Asima Shah:** Writing – review & editing, Validation, Supervision, Project administration, Investigation, Funding acquisition, Conceptualization. **Irfan Ahmad Raina:** Writing – review & editing, Writing – original draft, Methodology, Investigation, Formal analysis. **Asir Gani:** Writing – review & editing, Methodology, Formal analysis.

## Funding

This research was funded by Govt of India, Ministry of Science and Technology, Department of Biotechnology under grant no. BT/PR44635/NDB/39/792/2022 and FIST Life Sciences-Project and File No. SR/FST/LS-I/2019/588, dated 27 march 2021.

## Declaration of competing interest

The authors declare that they have no known competing financial interests or personal relationships that could have appeared to influence the work reported in this paper.

## References

[1] Kundangar M.R.D., Abubakar A. (2004). Thirty years of ecological research on Dal Lake. Kashmir. J. Res. Dev..

[2] Rather Z.A., Nazir R. (2015). Biochemical composition of selected macrophytes of Dal Lake, Kashmir Himalaya. J. Ecosyst. Ecograp..

[3] Syed F.N., Zakaria M.H., Bujang J.S., Christianus A. (2021). Functional properties, resistant starch and characterization of freshwater macrophytes. Int. J. Food Sci..

[4] Chen Q., Zhang J., Zhang Y., Kaplan D.L., Wang Q. (2022). Protein-amylose/amylopectin molecular interactions during high-moisture extruded texturization toward plant-based meat substitutes applications. Food Hydrocoll..

[5] Kumari B., Sit N. (2023). Comprehensive review on single and dual modification of starch: Methods, properties and applications. Int. J. Biol. Macromol..

[6] Jhan F., Gani A., Noor N., Ashraf Z.U., Gani A., Shah A. (2021). Characterisation and use of nano-reduced starch of underutilised cereals as a delivery system of folic acid through the human GI tract. Sci. Rep..

[7] Puri R., Gill B.S., Khetra Y. (2014). Influence of Acacia Gum, NaCl and sucrose on lotus stem starch physical properties. Int. J. Food Sci..

[8] Ahmad M., Gani A., Hassan I., Huang Q., Shabbir H. (2020). Mild alkali hydrolysis and ultra-sonication of starch nanoparticles. Sci. Rep..

[9] Chandak S.B., Dhull S.P., Bangar A.V., Rusu (2022). Cross-linking-Effects of the cross-linking on the physicochemical characteristics and film properties of lotus (*Nelumbo nucifera* G.) seed Starch. Foods.

[10] Z. A. Rather, P. Sharma, N. A. Dar, Analysis of macrophytes and their Nutrient content- a study of Dal Lake, Kashmir. J. Sustainability Biodiversity Conservation. 1 (2022) pp. 12-24.

[11] Lan X., Li Y., Xie S., Wang Z. (2015). Ultrastructure of underutilized tuber starches and its association with physicochemical properties. Food Chem..

[12] Gorecka K.D., Blaszczak W., Szwengiel A., Paukszta D., Lewandowicz G. (2014). Molecular and supermolecular structure of common cattail (*Typha latifolia*) starch. Starch/starke.

[13] Jhan F., Shah A., Gani A., Ahmad M., Noor N. (2020). Nano-reduction of starch in underutilised millets: Impact of structural, thermal, morphological and nutraceutical characteristics. Int. J. Biol. Macromol..

[14] Noor N., Gani A., Jhan F., Fazli A.A., Shah A., Huang Q. (2021). Nano-reduction of the lotus stem resistant-starch on functional and structural properties. ACS Food Sci. Technol..

[15] Awolu O.O., Olofinlae S.J. (2016). Production of water yam starch-based yoghurt Physico-chemical, functional and pasting properties of native and chemically modified water yam (Dioscorea alata) starch. Starch-Stärke.

[16] Jambrak R., Herceg Z., Šubarić D., Babić J., Brnčić M., Brnčić S.R., Gelo J. (2010). Ultrasound effect on physical properties of corn starch. Carbohydr. Polym..

[17] Vela J., Villanueva M., Ronda F. (2024). Ultrasonication: an efficient alternative for the physical modification of starches, flours and grains. Foods.

[18] AOAC. Official methods of analysis (15th ed.). New York, USA: Association of Official Analytical Chemists. Washington, D. C., USA (1995).

[19] Jan R., Saxena D.C., Singh S. (2016). Pasting, thermal, morphological, rheological and structural characteristics of Chenopodium (Chenopodium album) starch. LWT-Food Sci. Tech..

[20] Morrison W.R., Laignelet B. (1983). An improved colorimetric method for determining apparent and total amylose in cereal and other starches. J. Cereal Sci..

[21] Hsu L., Chen W., Weng Y.M., Tseng C.Y. (2003). Chemical composition, physical properties, and antioxidant activities of yam flours as affected by different drying methods. Food Chem..

[22] Abelti L., Teka T.A., Bultosa G. (2024). Physicochemical and structural characterization of starch prepared by using water lily (Nymphaea lotus), as food and non-food grade. Carbohydr. Polym. Technol. Appl..

[23] Tester R.F., Karkalas J., Qi X. (2004). Starch-composition, fine structure and architecture. J. Cereal Sci..

[24] Wang Q., Dong X., Ren F., Cui S.W. (2019). The impact of ultrasonic treatment on starch physicochemical properties: a review. Carbohydrate Poly..

[25] Kaur L., Singh J., Sandhu K.S., Singh N. (2012). Resistant starch–a review. Compr. Rev. Food Sci. Food Saf..

[26] Singh J., Kaur L., McCarthy O.J. (2003). Factors that affect the physicochemical, morphological, thermal and rheological characteristics of part of chemically modified starches applied to food-a review. Food Hydrocoll..

[27] Naseem S., Bhat S.U., Gani A., Bhat F.A. (2024). Starch exploration in Nelumbo nucifera and Trapa natans: Understanding physicochemical and functional variations for future perspectives. Int. J. Biol. Macromol..

[28] Jambrak R., Mason T.J., Lelas V., Herceg Z., Badanjak M. (2010). Ultrasonic physical, chemical and microstructural alteration of starch. Ultrason. Sonochem..

[29] Zhu F. (2016). Ultrasonic treatment of starch: effect on structural and physicochemical properties: review. Food Hydrocoll..

[30] Galazka V., Branyik T., Bednar P. (2014). Ultrasound industrial use. J. Food Sci. Technol. Int..

[31] Cui R., Zhu F. (2020). Ultrasound on the structural and physicochemical characteristics of sweet potato and wheat flours. Ultrason. Sonochem..

[32] Ahmed Z., Hussain A., Farooq U., Zhou Y., Ahmed I.A.M., Waseem M., Xu B., Manzoor M.F., Mugabi R. (2025). Multifrequency ultrasound treatments on structure, rheological and digestive properties of frozen wheat dough. Ultrason. Sonochem..

[33] Vela J., Villanueva M., Solaesa Á.G., Ronda F. (2021). Impact of high-intensity ultrasound waves on structural, functional, thermal and rheological properties of rice flour and its biopolymers structural features. Food Hydrocoll..

[34] Prakruthi L.Y., Krishnan H., Medha T., Kumarakuru K., Kumari P.V., Surekha C., Reddy C.K. (2025). Enhancing starch properties through dual modification: Ultrasonication and acetic acid treatment of non-conventional starches. Ultrason. Sonochem..

[35] Chang J.T., Chiu C.S., Chan Y.J., Liang Z.C., Lu W.C., Li P.H. (2025). Ultrasonication-induced structural and functional modification of starch in wheat flour. Ultrason. Sonochem..

[36] Rahaman A., Kumari X.A., Zeng M.A., Farooq R., Siddique I., Khalifa, Manzoor M.F. (2021). Ultrasound based modification and structural-functional analysis of corn and cassava starch. Ultrason. Sonochem..

[37] Lin L., Huang J., Zhao L., Wang J., Wang Z., Wei C. (2015). The influence of the granule size on the characteristics of lotus rhizome C-type starch. Carbohydrate Polym..

